# Awake minimally invasive parafascicular approach to a language eloquent brain tumour—surgical video

**DOI:** 10.1093/jscr/rjad519

**Published:** 2023-10-17

**Authors:** Miljyot S Sangha, Kapil M Rajwani, Sally-Ann Price, Hilary Wren, Ana M Pescador, Richard Gullan, Keyoumars Ashkan, Francesco Vergani, Ranjeev Bhangoo, Jose P Lavrador

**Affiliations:** Department of Neurosurgery, Kings College Hospital NHS Foundation, Denmark Hill, London, United Kingdom; Department of Neurosurgery, Kings College Hospital NHS Foundation, Denmark Hill, London, United Kingdom; Department of Neurosurgery, Kings College Hospital NHS Foundation, Denmark Hill, London, United Kingdom; Department of Neurosurgery, Kings College Hospital NHS Foundation, Denmark Hill, London, United Kingdom; Department of Neurosurgery, Kings College Hospital NHS Foundation, Denmark Hill, London, United Kingdom; Department of Neurosurgery, Kings College Hospital NHS Foundation, Denmark Hill, London, United Kingdom; Department of Neurosurgery, Kings College Hospital NHS Foundation, Denmark Hill, London, United Kingdom; Department of Neurosurgery, Kings College Hospital NHS Foundation, Denmark Hill, London, United Kingdom; Department of Neurosurgery, Kings College Hospital NHS Foundation, Denmark Hill, London, United Kingdom; Department of Neurosurgery, Kings College Hospital NHS Foundation, Denmark Hill, London, United Kingdom

**Keywords:** tubular retractor, minimal invasive surgery, glioma, awake surgery

## Abstract

Deep-seated brain tumours are surgically challenging to access. When planning approaches to these lesions, it is important to take into account eloquent cortical areas, grey matter nuclei, and subcortical white matter tracts. Traditionally, access to deep-seated lesions would require brain retraction; however, this is associated with secondary brain damage, which may impair neurological function. A trans-sulcal minimally invasive parafascicular approach allows gentle splitting of brain fibres and is thought to splay rather than sever white matter tracts. This is particularly important when approaching medially located, language-eloquent tumours, which lack brain surface expression. This video describes a minimally invasive approach to a deep-seated, language-eloquent brain tumour. We utilized preoperative cortical and subcortical planning to define a safe surgical corridor. We then demonstrate using intraoperative neuro-monitoring and mapping of the motor and language functions to define the boundaries of surgical resection. We find trans-sulcal minimally invasive parafascicular approach to be a safe and effective technique when approaching language-eloquent lesions medial to the main language subcortical networks.

## Introduction

Language eloquent lesions present specific challenges for surgical resection. Awake craniotomy techniques have revolutionized the treatment of both low and high grade gliomas when utilized alongside intraoperative cortical and subcortical mapping and monitoring.

Tumours located medial to the main association language network are particularly challenging to treat, as intraoperative mapping has a limited role (no role for cortical mapping). This results in preoperative mapping being vital in defining surgical corridors to safely access the lesions. Therefore, there is a need to improve and develop preoperative mapping techniques alongside surgical techniques that are minimally disruptive to the adjacent brain.

Trans-sulcal minimal invasive parafascicular (tsMIPS) approach has emerged as a promising surgical technique to approach deep-seated lesions. This approach allows preservation of the adjacent cortical and subcortical eloquent regions [1], whilst preventing secondary brain damage [2] as they splay rather than sever the white matter tracts (WMTs) [3].

In this video, we demonstrate a case using a tsMIPS awake craniotomy technique, for preservation of language and motor functions in a patient with a high-grade glioma.

## Case report

We describe a case of a 53-year-old, right-handed, gentleman who presented to the neuro-oncology service with a 10-day history of right-side facial weakness and word-finding difficulties. Imaging demonstrated a multifocal left-sided lesion, predominantly involving the left atrium. After patient and multidisciplinary input, a decision was made for a minimally invasive approach to the dominant deep-seated language eloquent brain tumour, medial to the main association tracts involved in language networks (Video 1). Navigated transcranial magnetic stimulation (nTMS) for motor and language mapping was used to define the external corridor, sulcal entry point, and preoperative tractography of language-eloquent tracts. The arcuate fasciculus (AF, orange), corticospinal tract (CST, yellow), inferior fronto-occipital fasciculus (IFOF, green), fronto-aslant (blue) and inferior longitudinal fasciculus tracts (purple) were used to plan the internal corridor approach.

A tsMIPS approach through the intraparietal sulcus medial to the main language network was performed. To limit injury to the WMTs, a tubular retractor (NICO BrainPath©) was used. The patient was woken up and his speech and motor function were assessed during brain cannulation.

Intraoperative neurophysiological monitoring was used throughout the procedure. Following the craniotomy, subdural strips of electrode were placed over both the primary motor and primary visual cortices to monitor motor and visual evoked potentials. Boundaries of our resection were determined by positive identification of language eloquent areas over IFOF (semantic errors, number 1) and AF (repetition errors, number 2) during subcortical electric stimulation using a low-frequency technique directly through the tubular retractor; and CST at 5 mA, using a high-frequency technique.

The procedure had no complications and the patient was discharged 48 hours post-operatively with stable motor and language function. The histological diagnosis confirmed a WHO Grade 4 Glioblastoma. Post-operative imaging demonstrated residual tumour infiltrating the WMTs and a minimal footprint left by the tsMIPS.

## Discussion

In this video, we demonstrated the use of both awake craniotomy and tsMIPS aid surgical resection of motor and language eloquent tumours medial to the main association language network. Together, these techniques can produce safe tumour debulking whilst preserving the functional status of the patient.

Preoperative cortical mapping is crucial in defining the external surgical corridor. We used the information provided by nTMS to select a sulcal entry point away from the motor eloquent area and positive nTMS responses for language [4]. The role of preoperative tractography is 2-fold: it firstly has a crucial role in surgical planning (internal corridor, avoid critical subcortical tracts) and secondly with patient counselling, particularly about the extent of resection and the risk for post-operative functional deficit [5]. Preoperative mapping can then be combined with tumour modelling to define the tsMIPS trajectory [6]. Trajectory visualization is critical in understanding and predicting the different modalities of mapping and monitoring we will use at different stages of surgery.

Another important point for discussion is the safety of brain cannulation in an awake patient. Despite a few episodes of coughing during the procedure, there were no complications related with the tubular retractor (misplacement or adjacent brain laceration). Utilizing a technique that allows for the patient to be awake is desirable as it allows for neurological functions at risk to be monitored during the cannulation.

A biopsy is the only other viable alternative for patients with similar lesions [7]. However, the growing evidence supports improved outcomes with prompt surgical resection. This means that techniques that increase the resectability of lesions with minimal neurological functional impacts are desirable. Further case series are necessary to fully validate the impact of such techniques.

Full informed consent has been taken from the patient. They are aware that this content will be shared on a website online.

## Supplementary Material

Minimally_invasive_approach_Video_rjad519
